# Synaptic Plasticity and Neurological Disorders in Neurotropic Viral Infections

**DOI:** 10.1155/2015/138979

**Published:** 2015-11-16

**Authors:** Venkata Subba Rao Atluri, Melissa Hidalgo, Thangavel Samikkannu, Kesava Rao Venkata Kurapati, Madhavan Nair

**Affiliations:** Department of Immunology, Institute of NeuroImmune Pharmacology, Herbert Wertheim College of Medicine, Florida International University, Miami, FL 33199, USA

## Abstract

Based on the type of cells or tissues they tend to harbor or attack, many of the viruses are characterized. But, in case of neurotropic viruses, it is not possible to classify them based on their tropism because many of them are not primarily neurotropic. While rabies and poliovirus are considered as strictly neurotropic, other neurotropic viruses involve nervous tissue only secondarily. Since the AIDS pandemic, the interest in neurotropic viral infections has become essential for all clinical neurologists. Although these neurotropic viruses are able to be harbored in or infect the nervous system, not all the neurotropic viruses have been reported to cause disrupted synaptic plasticity and impaired cognitive functions. In this review, we have discussed the neurotropic viruses, which play a major role in altered synaptic plasticity and neurological disorders.

## 1. Introduction

Over the years, Central Nervous System (CNS) has been shown to be the major target site for viral infections. Different viruses have different routes of entry and some viruses have been shown to penetrate the CNS (neuroinvasion) and can infect neurons and glial cells (neurotropism). Neurotropic viruses are categorized into neuroinvasive and neurovirulent groups and both of them are known to cause neuronal dysfunction. Interestingly, neuroinvasive virus is capable of accessing or entering the nervous system whereas neurovirulent virus is capable of causing disease within the nervous system. These neurotropic viruses such as coxsackie, Japanese, Venezuelan equine, and California encephalitis viruses, polio, mumps, echo, influenza, measles, and rabies cause acute infection. Other viruses that come under this category are members of the family Herpesviridae, such as Cytomegalo, Varicella-zoster, Herpes simplex, and Epstein-Barr viruses. The ones that cause a latent infection are Varicella-zoster and Herpes simplex viruses, whereas other viruses like measles, rubella, John Cunningham, and retroviruses such as human T-lymphotropic virus 1 and human immunodeficiency virus are also reported to be neuropathogenic [1]. All of these pathogens have different modes of entry into the human brain, causing the neuropathogenesis that leads to the neurocognitive disorders. However, the neuropathogenic mechanisms that are involved in these disorders neither are clear nor are elucidated yet and require further studies to identify the therapeutic targets. Neuropathogenic mechanisms that lead to these disorders need to be better understood to identify therapeutic targets.

Viral infections of the CNS that injure or destroy specific populations of brain cells are frequently associated with behavioral disturbances. These events occur either directly due to virus replication or indirectly as a result of the host immune response against the infectious agent. Neurotropic viruses can also persist in the CNS and, in the absence of cell destruction or inflammation, cause defects in goal-oriented behavior. Therefore, viruses may contribute to human CNS disorders whose etiology remains elusive. The finding of virally mediated impairment in neuronal function in the absence of cell destruction raises the possibility that noncytolytic viruses that persistently infect neurons may contribute to many human CNS disorders whose etiology is unknown. Since neurons are not destroyed by the viral infection, antiviral therapies resulting in viral clearance from these cells may restore normal brain function. Studies to test this hypothesis are currently underway.

Borna disease virus (BDV) is an enveloped virus with a nonsegmented, negative-strand RNA genome belonging to the Bornaviridae family within the Mononegavirales order. This neurotropic virus infects a wide variety of mammals, and serological evidence suggests that BDV, or a BDV-like virus, also infects humans. Infected hosts develop a wide spectrum of neurological disorders, ranging from immune-mediated diseases to behavioral alterations without inflammation, reminiscent of symptoms observed in human psychiatric diseases such as schizophrenia, mood disorders, and autism [2, 3]. BDV has a noncytolytic strategy of replication and primarily infects neurons of the limbic system, notably the cortex and hippocampus [4]. To date, the mechanisms responsible for the cognitive impairment of BDV-infected animals are still poorly understood. It is possible that neuronal infection by BDV impairs signaling pathways that are important for proper neuronal functioning and neuronal communication. Recently, it was observed that BDV specifically interferes with the activity-dependent enhancement of synaptic vesicle recycling, one component of neuronal communication as well as synaptic transmission [5]. Accordingly, in this review, we have discussed synaptic plasticity changes and neurological disorders in neurotropic viral infections, which affects neurocognitive functions.

## 2. Synaptic Plasticity

Plasticity is fascinating and one of the most important characteristics of the mammalian brain. Synapses have the ability to undergo lasting morphological and biochemical changes according to different and specific types of neuromodulators and stimuli, which forms a cellular basis for memory and learning. However, the relationship between a specific type of memory and the form of its synaptic plasticity is still unclear [6]. The responses that are involved can cause neural activity to have the capacity to modify neural circuit functions, which will give as a result different thoughts, behaviors, and feelings. This modification affects the efficacy or strength of synaptic transmissions and for more than a century has been thought to play a critical role in the brain capacity to integrate temporary involvements/feelings into stable traces of memory. In addition, it has been thought that synaptic plasticity played an important role in neural circuitry development. Evidence has demonstrated that certain prominent neuropsychiatric disorders happened as a consequence of impairments in synaptic plasticity mechanisms. Overall, many synaptic plasticity functions as well as mechanisms and forms have been described. Changes in enhanced or suppressed synaptic transmissions can have a temporal span of milliseconds to days or even longer [7].

## 3. Short-Term Synaptic Plasticity

Nearly every synapse studied in a variety of organisms, from invertebrates to mammals, has shown various forms of short-term synaptic plasticity which lasts for few milliseconds to a couple of minutes [8]. It is believed that these forms of synaptic plasticity play a significant role in short-term adaptations to transient changes in behavioral states, short-lasting forms of memory, and sensory inputs. The majority of these forms are produced by short outbreaks of activity that come as a result of a temporary buildup of calcium in presynaptic nerve terminals. This increment in calcium causes modifications in the possibility of neurotransmitter release by changing the biochemical processes that causes the exocytosis of synaptic vesicles [7]. Short-term synaptic plasticity was initially recognized as behaviorally significant in studies of marine organisms such as* Aplysia* [9]. One of the main effects of short-term synaptic plasticity is to act on the information processing function of synapses, allowing them to perform as filters with different properties.

## 4. Long-Term Synaptic Plasticity

**Figure 1:**
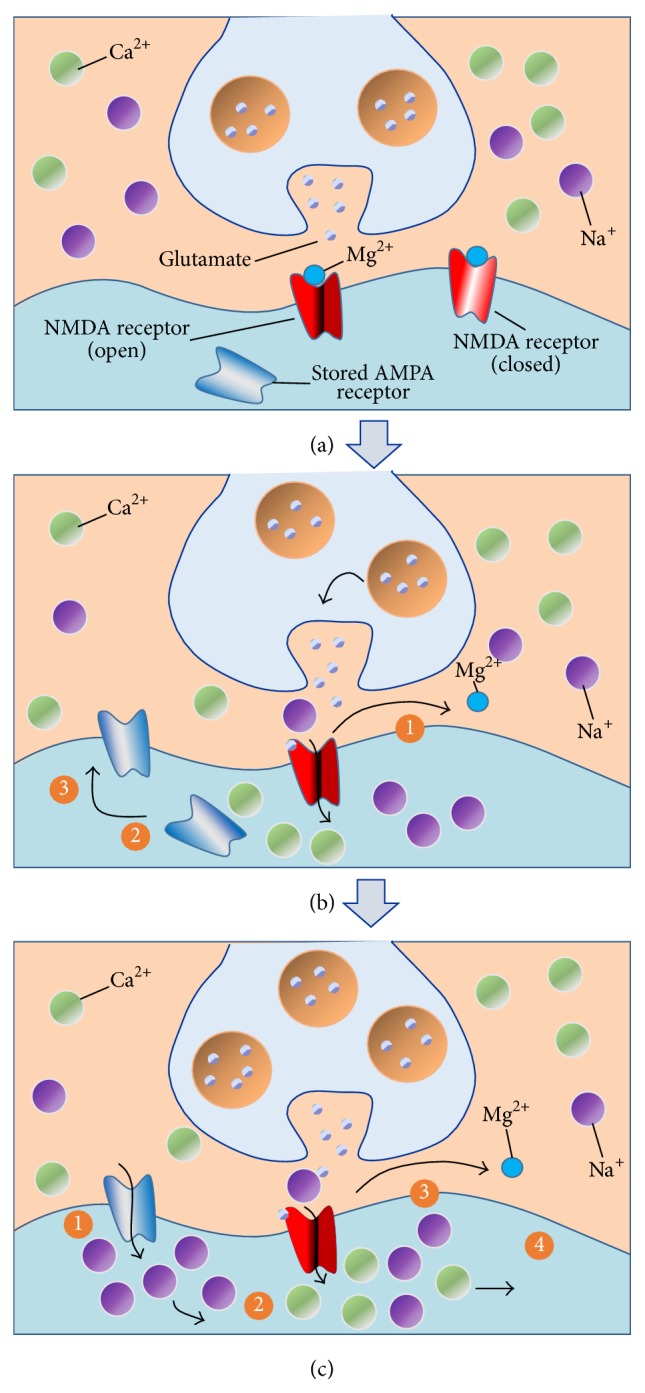
(a) Synapse prior to long term potentiation: NMDA and AMPA are two types of receptors at the postsynaptic neuron, for the neurotransmitter glutamate. NMDA receptors open in response to glutamate prior to potentiation. However, they are blocked by Mg^2+^. (b) Establishing LTP: NMDA receptors release Mg^2+^ after depolarization of the postsynaptic membrane in response to the activity. Na^+^ and Ca^+^ travel inside and induce the migration of internal AMPA receptors to the membrane. (c) Synapse exhibiting LTP: NMDA receptors are unblocked when depolarization is triggered by AMPA receptors. These two receptors are now responsible for action potentials.

**Figure 2:**
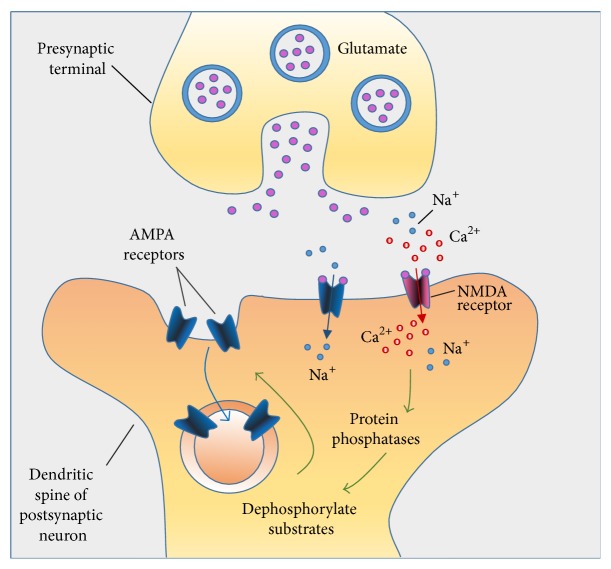
LTD mechanism: (a) Ca^2+^ ions enter in small quantities through NMDA receptors. (b) Activation of protein phosphatases. (c) Dephosphorylation of AMPA receptors leads to endocytosis of AMPA.

In the hippocampus, a repetitive stimulation of excitatory synapses is able to result in a potentiation of synaptic strength, lasting for hours to days, and it is referred to as long-term potentiation (LTP) or long-term synaptic plasticity. Different forms of long-term depression (LTD) are present in the majority of synapses that show LTP. LTD is an activity-dependent decline in the efficiency of neuronal synapses, resulting in a long patterned stimulus. Therefore, an important idea is that different patterns of activity are able to modify synaptic strength in a bidirectional way at excitatory synapses. Homeostatic plasticity has been recently recognized as an additional form of synaptic plasticity [10] as well as metaplasticity [11]. Schematic representation of the synapse (Figure 1(a)), establishing LTP (Figure 1(b)), and synapse exhibiting LTP (Figure 1(c)) was shown in Figure 1.  Figure 2 is showing the different mechanisms of long-term depression.

## 5. Rabies Virus

Rabies virus (RV) belongs to the Rhabdoviridae family and infects many animals (bats, skunks, foxes, and dogs) and human beings. RV in animal resides in salivary glands and spreads among different hosts via bites/scratches. RV infected animals can survive for years secreting virus particles in their saliva. In contrast to other infected animals, human infection results in fatal acute myeloencephalitis in untreated patients. By binding to acetylcholine receptors (nAchR) and neural cell adhesion molecules (NCAM), RV enters the axons of motor neurons at the neuromuscular junction [12]. Transneuronal spread occurs exclusively between synaptically connected neurons and the infection moves unidirectionally from postsynaptic to presynaptic neurons (retrograde spread). Once rabies infection reaches the CNS, marked behavioral and neurological symptoms begin and death almost always ensues [13]. In contrast to neuronal dysfunction related severe clinical manifestations, in postmortem examinations, only mild lesions in the CNS were observed. Various studies reported that fetal rabies causes neuronal dysfunction, including ion channel dysfunction and neurotransmitter abnormalities rather than neuronal damage [14–16], and downregulation of synaptic plasticity regulated protein has been reported in the silver haired bat rabies virus infection. Downregulation of these synaptic plasticity proteins leads to the blocked synaptic vesicle recycling, therefore, the reduced release and uptake of neurotransmitters [17]. Song et al. reported the decreased spine density in the street rabies virus infected hippocampus of mice and also reported that these changes were related to the depolymerization of filamentous actin (F-actin), a cytoskeleton protein that helps to regulate the morphogenesis and dynamics of dendritic spines [18].

## 6. Poliovirus

Poliovirus is part of the Picornaviridae family and enterovirus subgroup. The virus enters into the host and starts multiplying in the place of implantation, which is usually the gastrointestinal tract and pharynx. Before the onset of illness, the virus is present in the stool and throat. After the onset of the disease, the virus is less in the throat, but it will still be present in the stool for few weeks. Poliovirus is also capable of entering the bloodstream, invading lymphoid tissue, and subsequently infecting the CNS. Poliovirus was reported to enter the neurons by a receptor-mediated endocytosis at the neuromuscular junction and traverse from nerve terminal to the cell body using the host retrograde axonal transport system. It can hijack the host transport machinery using Tctex-1, a component of the dynein light chain involved in retrograde axonal transport [19, 20]. In* in vivo* intramuscularly injected mice with poliovirus, it was observed that trafficking of poliovirus in peripheral nervous system is difficult due to inefficient retrograde axonal transport and this could be the reason for low incidence of paralytic poliomyelitis in humans [21]. It replicates in motor neurons of the brain stem and the anterior horn, which leads to poliomyelitis and cell destruction. About 95% of the infected patients are asymptomatic and nearly 4%–8% of cases show a nonspecific but minor disease that does not invade the CNS. In few days after a prodrome, nonparalytic aseptic meningitis happens in 2% of infected patients, whereas flaccid paralysis happens in less than 1% of infections. After prodromal symptoms, paralytic symptoms begin and these symptoms usually progress for the next 3 days. Usually, when the temperature goes back to standard levels, there is no additional paralysis.

Of all poliomyelitis survivors, only 25% showed a relapse of fatigue, weakening of the muscles, or paralytic symptoms, and this is referred to as postpolio syndrome. It has been suggested that the cause of this syndrome is a reappearance of latent virus. However, there is no evidence yet to support this argument [22]. After the initial acute infection, there are some recovery mechanisms that occur in a short period of time. Patients can experience temporary paralysis, followed by a partial paralysis recovery. This temporary paralysis could be happening as a consequence of temporary silencing of neurons by transient virus infection, neuronal response to transient inflammation or to transient release of inhibitory neuroactive agents. Another probability would be when local plasticity allows replacement of lost motor neurons by cytolytic virus infection with uninfected neurons. The mechanisms behind the loss of motor neurons are still unclear. An understanding of this mechanism may give us an insight of postpolio syndrome as well as other motor neuron illnesses [23].

Some data found in poliomyelitis cases demonstrated that redundant systems and cells take over when loss of neurons occurs. This is one of the main characteristics of neurological diseases. However, there is a certain limit to the amount of neurons that can be lost. Therefore, after a certain percentage, symptoms only start to be more evident. For instance, when dopamine neurons are lost in Parkinson's disease, it leads to muscle rigidity and tremor instead of the flaccid paralysis seen in PV. Narcolepsy is another example, in which hypocretin neurons are lost. This loss results in a temporary flaccid paralysis during cataplexy. Positive correlation has been observed between the severity of symptoms and the number of neurons lost. However, asymptomatic cases are seen if there is less than 50% loss of neurons. In this perspective, the combination of initial killed cells by PV and the loss of neurons as a consequence of aging may lead to the manifestation of postpolio syndrome. As a result, there would be a high probability of insufficient motor neurons to continue with normal functions on the affected muscles. An alternative could be to target motor neurons with a low-grade autoimmune mechanism when they are PV-initiated [23].

## 7. Japanese Encephalitis

Japanese encephalitis (JE) is caused by the JE virus (JEV), which is a positive-sense and single-stranded RNA virus that forms part of the Flaviviridae family. The transmission of this virus occurs in a zoonotic way between water birds, pigs, and mosquitoes. Humans are a dead end host because of low level and transient viremia after being infected accidentally [24]. This disease is most commonly seen in Southeast Asia, where it affects about 50,000 individuals and causes approximately 10,000 deaths per year. In new endemic areas, both children and adults are found to be affected and, on the other hand, mostly children are affected in regions where this infection has been endemic for several years. In few areas, such as Korea and Japan, this virus has been controlled for a long time by immunization. In these areas, the virus may affect the elderly only. Case studies reported that children with JE have severe encephalitis characterized by a high frequency of seizures, deep coma, and mortality rates. In addition, seizures are most commonly found in 64% to 80% of children in comparison to only in 10% of adults [25–27].

The possible route of CNS entry of JEV is through the capillary endothelial cells (CEC), as the entry between CECs is inhibited by tight junctions [28]. In an* in vivo* mouse model of intravenous JEV infection, it was shown that viral titers increased exponentially in the brain (propagates in neurons) 2–5 days after infection that led to the exponential increase in the inflammatory cytokines and chemokines in the brain. Increased blood-brain barrier (BBB) permeability was observed only after 4th day of postinfection [29]. Most individuals that survived JEV infection experience severe neurological sequelae, such as language and cognitive impairments, motor deficits, and learning difficulties. In JE individuals, neuronal death can be caused by either the virus or as a bystander method facilitated by a strong inflammatory attack and microglial activation [30, 31]. Neuronal loss is regulated by the CNS by inducing the differentiation of new astrocytes and neurons from inhabitant multipotential neural progenitors cells (NPCs) [32]. These NPCs have the ability to self-renew over their lifespan and are located in neurogenic zones such as the dentate gyrus of the hippocampus and the subventricular zone (SVZ) [33]. Active NPCs are vastly lost from the SVZ by inhibiting their cycling ability as a result of JEV infection. Therefore, the formation of neurospheres by SVZ cells is greatly affected when they are JEV infected. The critical postnatal age is a predominant target and decreases the NPCs population in the SVZ and damages the recovery process. These might have a critical effect in JE survivors and their neurological outcomes [34]. JEV-infected microglia secretes inflammatory molecules that cause death of bystander neurons. Certain proinflammatory cytokines such as IL-6, TNF-*α*, and ROS/NO and MCP-1 are secreted in high concentrations by the infected microglia [35]. Secretions in high levels of these factors are antineurogenic and neurotoxic [36, 37].

## 8. Influenza Virus

Influenza is a serious health concern and economic burden since it remains as the primary cause of disease and death worldwide. Even though a lot of individuals recover from this infection, the short- and long-term effects on the CNS remain unclear. Cognitive and neurological consequences related with this virus have been described for many decades after the 1918 “Spanish” flu, as well as during the pandemic of influenza A H1N1. However, mechanisms associated with the symptoms are still unclear [38–41]. Most influenza strains are nonneurotropic, including the ones responsible for pandemics [42–44]. This suggests that neurological symptoms do not happen as a result of direct CNS viral infection but because of a neuroinflammation that came from an induced peripheral viral infection.

The peripheral innate immune system has been reported to get activated, producing certain cytokines such as interleukin-1*β* (IL-1*β*), IL-6, and TNF-*α* within the brain. As a result, this activation can have deleterious effects on emotional and cognitive behavior [45–48]. Long-term potentiation can be directly impaired and neurotrophins inhibited [49] by inflammatory cytokines [50, 51]. Neurotrophins are important for memory formation, synaptic plasticity, and neuronal function and survival [52–54]. Also, hippocampal neuronal morphology alterations occur after central and peripheral administration of lipopolysaccharide (LPS) takes place, inducing an innate immune response [55, 56]. While spine density and dendritic branching changes have an effect on synaptic plasticity [57, 58], induced inflammation alterations in neuronal complexity result in a hippocampal function deficit related to memory and learning. Infected mice with influenza A/PR8/34 (H1N1) were observed to have cognitive deficits and hippocampal neuroinflammation that were related to substantial changes in dentate gyrus neuron morphology and CA1 as well as the loss of neurotrophic factors [59].

## 9. Herpes Simplex

Herpes simplex virus (HSV) is a double stranded DNA virus. It was shown to enter the brain amygdala and hippocampus through the olfactory nerve and locus coeruleus. It has the tendency to enter latency within the CNS. In the infected mice model, both primary infection and reactivation of latent DNA in the brain led to neuronal damage that resulted in loss of memory, learning deficits, and behavioral change [60, 61]. In addition, it is transported transsynaptically, anterogradely, and retrogradely. HSV infection of the CNS can be lethal by affecting the inferior and medial temporal lobe. Some of the symptoms seen in acute Herpes simplex encephalitis are Wernicke's aphasia, headache, fever, epileptic seizures, confusion, and low consciousness. Memory impairment may persist when the limbic system and temporal lobe are affected [62].

The viral DNA was found in very few young people and children's brains in comparison to elderly brains [63, 64], which may prove that HSV1 enters into older people's brain as a result of a weakened immune system. In addition, 60% of patients who are carriers of the APOE-e4 allele show a higher risk factor for Alzheimer's disease (AD) when the virus is present [65]. It has also been reported that HSV1 can be reactivated in brain, producing a recurrent infection [63]. Most of the time, HSV1 results in cell death. As a result, it was suggested that HSV1 might be reactivated during stress, inflammation, or immunosuppression conditions which may lead to the neuronal damage and subsequently to the development of AD, especially in APOE-e4 carriers. Neuropathological processes in case of HSV1 acting with APOE-e4 might occur due to the accumulation of AD-like tau (P-tau) and beta amyloid (A*β*) [63, 65]. Reactivation events are known to occur in the peripheral nervous system. HSV1 is located in the trigeminal ganglia, where it causes an evident damage by the appearance of cold sores in approximately 40% of infected individuals.

## 10. Varicella-Zoster

Varicella-zoster virus (VZV) is a human alphaherpesvirus that infects up to 90% of the human population. Following primary infection (varicella or chicken pox) which is more common during childhood, the virus establishes a lifelong latent infection in the dorsal root ganglia of the host and it may cause neurological complications such as postherpetic neuralgia (PHN), zoster-associated pain (ZAP), encephalitis, segmental motor weakness, myelitis, or arteritis, which may be fatal or may be followed by significant morbidity [66–70]. The main clinical characteristics of Herpes-zoster are dermatomal rash, acute pain, and neurologic symptoms [71]. Encephalitis and meningitis have also been observed to be caused by VZV [72]. The CNS complications can occur during primary infection and in the reactivation of VZV. The more serious complications occur when VZV invades the spinal cord or cerebral arteries after reactivation of the virus. The most common complication in 7 to 35% of infected individuals is PHN. Its symptoms involve constant, severe, stabbing or burning, dysesthetic pain. Although pathogenic mechanisms of PHN are unknown, two possible mechanisms are altered excitability of ganglionic or spinal cord neurons and persistent or low-grade productive virus infection in ganglia [73–75]. It has been observed that primary VZV infection causes VZV to be persistent in dorsal root and cranial nerve ganglia [71, 76–78]. When reactivation of VZV occurs, the feature dermatomal rash of Herpes-zoster takes place due to the movement of VZ virions through neuronal cell bodies into the skin. Weakness or paralysis of ipsilateral facial muscles is caused due to the zoster infection of the seventh cranial nerve (geniculate) ganglion [79]. Lower motor neuron type weakness in the arm and leg is caused by the cervical or lumbar distribution of zoster, respectively [80, 81].

## 11. Cytomegalovirus

Cytomegalovirus (CMV) is a common intrauterine pathogen that causes congenital developmental abnormalities of the CNS and developmental neurological disabilities such as CMV encephalitis, characterized by focal areas of reactive gliosis, reactive mononuclear cells, microglial nodules, and ventriculoencephalitis [82]. In the immunocompromised patients, CMV was reported to reach the brain from the blood and disseminated further by the CSF prior to the subsequent movement into the brain parenchyma [83]. It has been observed to be a lethal ventriculoencephalitis in individuals with advanced AIDS [84]. It has also been reported that CMV infects more cells in the subventricular and ventricular areas of the brain in congenital infected adults and children [84–86]. These results have also been observed in congenital CMV mouse models [87]. Impairment of neural stem cells (NSCs) may result in neuropathological effects that are related to CMV brain infection [88]. Currently, the leading cause of childhood disorders as well as birth defects in the United States is the congenital CMV infection. Every year, about 8,000 children show some neurological sequelae that are associated with congenital CMV infection. Nevertheless, the neuropathogenesis of this infection is still unclear. Human neural precursor cells have been reported to be vulnerable to CMV infection [89–91]. Alteration of the cellular differentiation process of these cells is observed in the presence of CMV infection [91, 92]. In CMV infected mice models, the expression of immediate early (IE) genes was held in the postnatal infected brain cortex. This might have happened because of the development of infected NSCs [93]. Likewise, IE expression in the cerebellum is related to the late development and movement of precursor cells [94]. A better understanding of the relationship between NSCs and CMV is critical for the development of neuropathogenic mechanisms of viral infection.

## 12. Epstein-Barr Virus

Epstein-Barr virus (EBV) is a human herpesvirus related to epithelial and lymphoid malignancies. This virus causes transmissible mononucleosis. Occasionally, EBV is said to produce an extensive variety of CNS infections, such as Guillain-Barre syndrome, Bell Palsy, transverse myelitis, cerebellitis, aseptic meningitis, and encephalitis [95–98]. These neurological complications occur during primary infection, typically in childhood. For the first time, role of EBV in the development of multiple sclerosis was reported by Fraser et al. [99]. Multiple sclerosis is a chronic demyelinating disease of the CNS causing axonal pathology and episodic or progressive neurological disability [100]. The role of EBV in the pathogenesis of multiple sclerosis could be due to the molecular mimicry between EBV and CNS antigens that results in immunological cross-reaction and resultant autoimmune damage in the CNS [101]. There is a strong correlation between the frequency of CD8+ T cells and EBV infected B cells in the CNS and it indicates that immunological response found in the multiple sclerosis is primarily against EBV, with bystander damage to the CNS [102, 103].

## 13. Human T-Lymphotropic Virus 1

Human T cell leukemia virus type 1 (HTLV-1) is a type C retrovirus. Even though most of infected patients do not show any symptoms, HTLV-1 is responsible for adult T cell leukemia (ATL) and HTLV-1-associated myelopathy/tropical spastic paraparesis (HAM/TSP), which is a progressive demyelinating disorder [104]. HAM/TSP is a progressive and chronic inflammatory illness. In HAM/TSP, a deprivation of white matter can be observed inside the lateral funiculi spinal cord in the lumbar and thoracic tissue segments. The brainstem and cervical spinal cord have also shown degeneration, even though this might have happened as a result of Wallerian degeneration [105, 106]. The primary region of neuronal damage was found inside the corticospinal tract. Patients who exhibited this damage reported weakness in their lower limbs [107]. HAM/TSP is usually present as a spastic paraparesis. Sexual and urinary dysfunctions as well as lower back pain are some of the common symptoms [104, 108, 109]. HAM/TSP can be divided into two phases. The first phase is seen as an inflammatory response and the second phase as a chronic degenerative stage [110]. The inflammation seen in the first phase affects the BBB and lymphocyte trading into the CNS increases its chances of happening [111–114].

CNS cell loss and demyelination, occurring in HAM/TSP individuals, could involve different mechanisms. Some of these are the autoimmune mechanism of molecular mimicry, the direct damage mechanism, and the bystander mechanism [115]. HTLV-1-associated pathogenesis inside the CNS could be related to an autoimmune mechanism that includes molecular mimicry. In addition, the direct damage mechanism involves the infiltration of activated CD8+ cytotoxic T lymphocyte cells specific for HTLV-1 Tax protein. This indicates a continuous manifestation of viral proteins or replicating virus [116]. In this case, cellular damage occurs from the release of inflammatory molecules and the directed lysis of infected cells. The bystander mechanism involves the release of proinflammatory cytokines in response to HTLV-1, which causes damage in the CNS [117]. Proinflammatory cytokines such as interferon-*γ* (IFN-*γ*) and TNF-*α* are proposed to cause loss and dysfunction of CNS cells as well as disruption of the BBB [118].

Even though neurons are not proposed to harbor virus* in vivo* [119], HTLV-1 neuronal infection demonstrated the potential to do so* in vitro* by neuroblastoma cell line infection and nontumorigenic origin neuronal cell line, such as HCN-1a and HFGC [106]. As mentioned above, another proposed mechanism is the autoimmune pathology of molecular mimicry. This mechanism involves the recognition of a host antigen as a viral protein by the immune system.

## 14. Human Immunodeficiency Virus

Human immunodeficiency virus (HIV) is a neurotropic virus that goes into the brain briefly after the infection [120]. HIV causes neurotoxic and inflammatory host responses by replicating in brain microglia and macrophages. HIV infection can also lead to neurological disorders known as HIV-associated neurocognitive disorders (HAND). Motor, behavioral, and cognitive abnormalities can be observed in HAND. HIV-1 is classified into three groups (M, O, and N) and into nine genetic subtypes (A–K). Among these, clades B and C are the most circulating HIV-1 variants (>86%) [121] worldwide. In North America, Australia, and Western Europe, the leading one is clade B, whereas, in Latin America, Africa, and Asia, the most common one is clade C. Before the use of highly active antiretroviral therapy (HAART), 30% of advanced HIV-1 infected individuals showed HIV-associated dementia (HAD) symptoms [122, 123]. On the other hand, Satishchandra et al. (2000) [124] along with other studies [125] stated a very low frequency of HAD in about 2% of patients that were HIV-1 clade C infected from India. After the introduction and use of HAART worldwide, the frequency of HAD has reduced significantly. However, 40–50% of patients still show symptoms related to HAND [126–130]. HIV is transported by infected perivascular macrophages and monocytes through the BBB. A decreased neuronal function and plasticity were observed in postmortem brains of HAND patients. These can be seen at systemic and cellular levels. At the cellular level, HAND patients showed a decreased dendritic and synaptic density as well as a synaptodendritic damage [130], which can cause a neural network interruption and eventually lead to caspase-3-dependent neuronal apoptosis [131]. This can be observed at the system level as white and grey matter degeneration in cortical and subcortical areas [120, 132]. The basal ganglia are mainly affected [133, 134]. Recently, we have reported dysregulated synaptic plasticity genes expression in clade B infected SK-N-MC neuronal cells and clades B and C infected astrocytes. We have observed induced apoptosis and decreased spine density in clade B infected neuronal cells compared to clade C infected and control cells. These observations indicate that HIV-1 clade B is more neuropathogenic than clade C [135]. In the process of exploring the epigenetic regulation of synaptic plasticity genes expression in HIV infected neuronal cells, we have observed HDAC2 upregulation in these cells. Inhibition of HDAC2 by using the vorinostat resulted in the recovery of synaptic plasticity genes expression in HIV infected neuronal cells [136]. In HIV infection, the leading cause of reduced neuronal function may be due to the synaptodendritic injury rather than neuronal loss. Furthermore, a difficult issue for neuro-AIDS is the number of HIV-positive individuals that abuse illicit drugs. Heroin abuse is a major risk factor for HIV transmission, while abuse of stimulants has become one of the risk factors for HIV. Alcohol and other drugs of abuse cause oxidative stress to increase as well as brain atrophy and bad performance in neurocognitive assessments [137].

## 15. HIV Induced Neuroinflammation and Neurotoxicity

A better understanding of the cellular and molecular mechanisms of HIV neurotoxicity is required for the prevention of HIV neuropathology. HAND individuals usually experience prolonged symptoms of HIV encephalitis. In this neuroinflammatory condition, the presence of HIV-infected microglial cells, multinucleated giant cells, myelin loss, development of microglial nodules, and astrogliosis is observed [138–140]. When microglia, macrophages, and distressed astrocytes get activated, the uptake of excitotoxic neurotransmitters is reduced, inhibiting plasticity [141]. As a result, the formation of dendritic synapses and spines is also reduced. Furthermore, neuronal survival is compromised when the release of IL-1*β*, TNF-*α* [142], and CXCL12 [143] by infected glial cells takes place [144–146]. Therefore, glial cells have the capacity to decrease homeostasis-mediated plasticity by promoting or exacerbating HIV-induced neurotoxicity.* In vitro* data have demonstrated that cytokines can encourage neuronal loss. Nevertheless, microglia's role in HIV neuropathology is still unclear, as microglia can also be activated by dying and distressed neurons. Additionally, the basal ganglia exhibit a selective susceptibility to synaptodendritic injury that cannot be described only by inflammatory cytokines. All these data support the idea that HIV stimulates the release of different viral proteins and soluble host cell-derived factors that may collaborate to cause the pathology of synapses.

## 16. Role of HIV Proteins in Neurotoxicity

Out of nine HIV proteins reported to cause neuronal injury, the transactivator of transcription (Tat) protein is one of the major viral proteins that is able to cause neurotoxicity. Tat is vital for HIV replication and influences transcription initiation and elongation [147] at the HIV promoter. In addition, Tat can reduce neuronal survival by different mechanisms, such as inflammatory cytokine [148], impairment of mitochondrial function [149], and activation of ionotropic glutamate receptors [150]. HIV-infected cells can release Tat [151] and it has been observed that the combination of HIV-1 clade B and Tat protein intensifies the production of reactive oxygen species and inhibits redox expression compared to clade C or its Tat protein. These data show that HIV-1 clades B and C produce different effects of thiol alteration and redox expression. In addition, HIV-1 clade B induces oxidative stress, which leads to more immunoneuropathogenesis than HIV-1 clade C [152]. Recently, we have reported that clade B Tat differentially regulates the synaptic plasticity genes expression compared to clade C [153]. While penetration of antiretroviral drugs across the blood-brain barrier might be crucial for the treatment of HAND, we are using the nanotechnology based approach to inhibit HIV infection and latency in the CNS cells by transferring the anti-HIV drugs coupled with vorinostat [154]. Nef, Vif, Vpr, and Vpu are key accessory proteins in HIV pathogenesis that affect some host cell functions, such as cytoskeleton contraction [155], and promote the release of virions and optimize viral replication [156]. Once these proteins are released, they can induce neuronal apoptosis [157] throughout different mechanisms, such as the activation of caspase-8 (Vpr) and formation (Vpr and Vpu) or direct binding (Nef) to ion channels. This will lead to lethal abnormal membrane depolarization [158].

**Figure 3:**
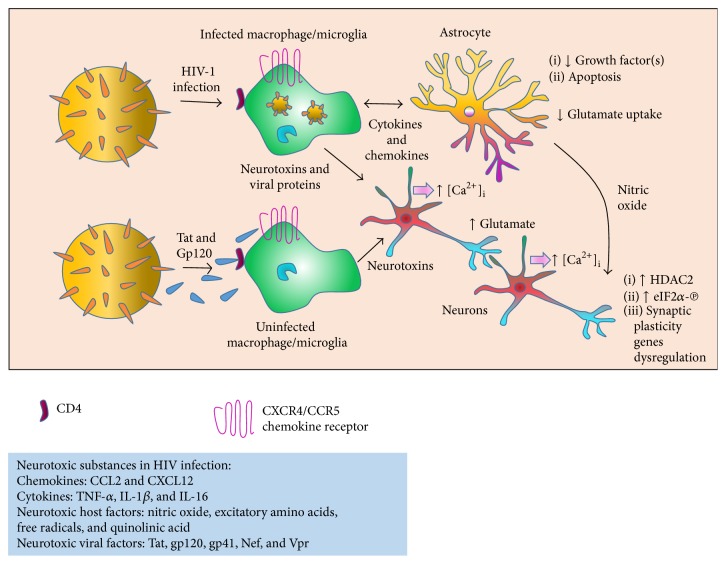
A model of HIV-induced neurotoxicity. Infected microglia or macrophages release viral proteins, chemokines, and cytokines. This activates uninfected microglia and macrophages. Neuronal injury, synapse damage, and cell death occur because immune activated and HIV-infected brain microglia and macrophages release neurotoxic elements. Excessive influx of Ca^2+^ ions occurs because of the overactivation of NMDA receptor-coupled ion channels that mediate neuronal injury. As a consequence, potentially harmful enzymes, release of glutamate, and free-radical formation are triggered. Subsequently, glutamate overstimulates NMDA receptors on nearby neurons, which causes additional injury. Upregulation of HDAC2 expression and increased eIF2*α*-phosphorylation leads to the dysregulated synaptic plasticity gene and protein expression which results in impaired synaptic plasticity.

Glycoprotein gp120 is another structural protein that has been reported to induce neuronal apoptosis. This gp120 has a significant function in the viral infection cycle and binds to chemokine coreceptors CCR5 and CXCR4, allowing conformational change and entering of the virus into cells [159]. Neuronal apoptosis can be induced by a short exposure of neurons to gp120 [160, 161]. It has also been reported to stimulate axonal degeneration [162] as well as dendritic injury [163, 164]. These two main effects are associated with the synaptodendritic atrophy seen in HAD [165]. Gp120 transgenic mice have been reported to show dendritic reduction and neuronal loss [166], which indicates that gp120 is capable of reducing and affecting synaptic plasticity.  Figure 3 is showing the role of HIV infection, Tat, and gp120 in the HIV-induced neurotoxicity.

In neuronal diseases including neurotropic viral infections, the peripheral innate immune system has been reported to get activated, producing certain cytokines within the brain. As a result, this stimulation can have deleterious effects on synaptic plasticity, emotional, and cognitive behavior. While current research in this area is ongoing, the role of synaptic plasticity during neurotropic viral infections and associated neurodegenerative diseases are the most recent and least understood. While there is an agreement that many neurodegenerative diseases are characteristic of a vigorous inflammatory response, it remains unclear how this process is related to disease processes. The interaction of viruses with their hosts is remarkable in numerous ways. The interaction between virus and host* in vivo*, especially in brain, is very complicated by the categorized arrangement of cells, tissues, and systems, which offer the appropriate protective response. If this united response to viral infection is not sufficient, then there is every possibility of resulting in disorders associated with synaptic plasticity and cognitive effects. Damage to brain cells can result from viral replication or by the action of the activated immune system and may result in the death of neuronal cells. Designing methods such as live-cell and intravital imaging together with neuron culturing methods supplemented by the capability to construct recombinant viruses will enable researchers to study some of the fundamental characteristics of virus replication as well as spread within and between neurons. These methodologies will empower the scientists to understand the mechanisms of how neurotropic viruses get entrance to and spread in the brain. New methods, such as deep sequencing of viral nucleic acid from clinical samples or single-molecule sequencing will enable identification of more neurovirulent and/or neuroinvasive virus mutants and will provide a genetic view of host barriers and viral bypass mechanisms and will help in improving the other cognitive associated neurodegenerative disorders.
